# Supplementary material for: The resistomes of rural and urban pigs and poultry in Ghana
Source: mSystems. 2023 Sep 22;8(5):e00629-23. doi: 10.1128/msystems.00629-23 (PMC10654090; doi:10.1128/msystems.00629-23)
Supplement: Supplemental tables and figures — Tables S1 to S11 and Figures S1 to S7. [file msystems.00629-23-s0001.docx]

**Supplementary material**

**Table S1:** Description of the pig fecal samples

| **No.** | **Sample ID** | **Production system** | **Characteristics of the site** | **Biosecurity Level** | **Community** | **District** | **Region** | **Zone** |
| --- | --- | --- | --- | --- | --- | --- | --- | --- |
| 1 | AS54 | Semi-intensive | Rural setting with mud housing- farm near households. | Low End | Soe | Bolgatanga | Upper East | Northern |
| 2 | CA63 | Semi-intensive | Rural setting with mud housing-Farm near households | Low End | Soe Zuboko | Bolgatanga | Upper East | Northern |
| 3 | MM36 | Intensive | Urban setting with concrete housing-farm isolated from settlement | High End | Luho | Wa | Upper West | Northern |
| 4 | PB61 | Intensive | Urban setting with concrete housing-farm isolated from settlement | High End | Siriyiri | Wa | Upper West | Northern |
| 5 | JKA41 | Semi-intensive | Urban setting with concrete housing-farm isolated from settlement | High End | Katariga | Sagnerigu | Northern | Northern |
| 6 | TYT24 | Intensive | Urban setting with concrete housing-farm isolated from settlement | High End | Katariga | Sagnerigu | Northern | Northern |
| 7 | CON40 | Semi-intensive | Rural setting with concrete housing- farm isolated from settlement | Low end | Kokosua | Ayensuanu | Eastern | Southern |
| 8 | ANY42 | Intensive | Rural setting with wooden and concrete housing- farm isolated from settlement | Low end | Kokosua | Ayensuanu | Eastern | Southern |
| 9 | KF52 | Semi-intensive | Urban setting with concrete housing-farm isolated from settlement | Low End | Amanokrom | Akwapim North | Eastern | Southern |
| 10 | EOA39 | Intensive | Urban setting with concrete housing-farm isolated from settlement | High End | Bonkua | Ayensuanu | Eastern | Southern |
| 11 | SC34 | Semi-intensive | Rural setting with concrete housing- farm near households | Low End | Amanokrom | Akwapim North | Eastern | Southern |
| 12 | KN57 | Intensive | Urban setting with concrete housing-farm isolated from settlement | High End | Obosomase | Akwapim North | Eastern | Southern |
| 13 | VG62 | Intensive | Urban setting with concrete housing-farm isolated from settlement | High End | Adjei Kojo | Ashiaman | Gt.Accra | Southern |
| 14 | AK47 | Intensive | Urban setting with concrete housing-farm isolated from settlement | High End | Adjei Kojo | Ashiaman | Gt.Accra | Southern |
| 15 | DL54 | Intensive | Urban setting with concrete housing-farm close to settlement | High End | Sagoanah | Ladma | Gt.Accra | Southern |
| 16 | BAK63 | Semi-intensive | Urban setting with wooden housing-farm isolated from settlement | Low End | Labadi | Ladma | Gt.Accra | Southern |
| 17 | JKY71 | Intensive | Urban setting with wooden and concrete housing-farm isolated from settlement | High End | Inchaban Nkawnta | Shama | Western | Southern |
| 18 | FKA76 | Semi-intensive | Urban setting with wooden and concrete housing-farm close to settlement | Low End | Inchaban Nkawnta | Shama | Western | Southern |
| 19 | LA74 | Intensive | Rural setting with concrete housing- farm isolated from settlement | High End | Onwe | Ejisu | Ashanti | Middle |
| 20 | NND65 | Intensive | Rural setting with concrete housing- farm isolated from settlement | Low End | Onwe | Ejisu | Ashanti | Middle |
| 21 | EJ37 | Semi-intensive | Rural setting with wooden housing- farm close to settlement | High end | Tuobodom | Techiman North | Bono East | Middle |
| 22 | AK32 | Semi-intensive | Rural setting with wooden housing- farm isolated from settlement | Low end | Tuobodom | Techiman North | Bono East | Middle |
| 23 | BB27 | Intensive | Rural setting with concrete housing- farm isolated from settlement | High end | Bamiri | Techiman South | Bono East | Middle |
| 24 | JY65 | Semi-intensive | Rural setting with concrete housing- farm isolated from settlement | Low end | Bamiri | Techiman South | Bono East | Middle |
| 25 | EAB56 | Intensive | Rural setting with concrete housing- farm close to settlement | High End | Abisim | Sunyani | Bono | Middle |
| 26 | SAY52 | Intensive | Rural setting with concrete housing- farm isolated from settlement | Low End | Abisim | Sunyani | Bono | Middle |
| 27 | AT52 | Semi-intensive | Urban setting with concrete housing-farm close to settlement | Low End | Fiapre | Sunyani West | Bono | Middle |
| 28 | EK55 | Semi-intensive | Rural setting with concrete housing- farm Isolated from settlement | Low End | Domasua | Sunyani West | Bono | Middle |
| 29 | TO52 | Intensive | Rural setting with concrete housing- farm Isolated from settlement | Low End | Besease | Atwima | Ashanti | Middle |
| 30 | ST37 | Semi-intensive | Rural setting with concrete housing- farm Isolated from settlement | Low End | Nerebehi | Atwima | Ashanti | Middle |

**Table S2:** Description of the poultry caecal sample sites

| **No.** | **SampleID** | **Characteristics** | **Location of site** | **Region** | **Zone** |
| --- | --- | --- | --- | --- | --- |
| 1 | ASH1 | Rural free-range birds from live bird markets | Ashiaman | Greater Accra | Southern |
| 2 | ASH2 | Rural free-range birds from live bird markets | Ashiaman | Greater Accra | Southern |
| 3 | ASH3 | Rural free-range birds from live bird markets | Ashiaman | Greater Accra | Southern |
| 4 | ASH4 | Rural free-range birds from live bird markets | Ashiaman | Greater Accra | Southern |
| 5 | ASH5 | Rural free-range birds from live bird markets | Ashiaman | Greater Accra | Southern |
| 6 | ASH6 | Rural free-range birds from live bird markets | Ashiaman | Greater Accra | Southern |
| 7 | ASH7 | Rural free-range birds from live bird markets | Ashiaman | Greater Accra | Southern |
| 8 | ASH8 | Rural free-range birds from live bird markets | Ashiaman | Greater Accra | Southern |
| 9 | ASH9 | Rural free-range birds from live bird markets | Ashiaman | Greater Accra | Southern |
| 10 | ASH10 | Rural free-range birds from live bird markets | Ashiaman | Greater Accra | Southern |
| 11 | ADA1 | Rural free-range birds from live bird markets | Ada | Greater Accra | Southern |
| 12 | ADA2 | Rural free-range birds from live bird markets | Ada | Greater Accra | Southern |
| 13 | ADA3 | Rural free-range birds from live bird markets | Ada | Greater Accra | Southern |
| 14 | ADA4 | Rural free-range birds from live bird markets | Ada | Greater Accra | Southern |
| 15 | ADA5 | Rural free-range birds from live bird markets | Ada | Greater Accra | Southern |
| 16 | ADA6 | Rural free-range birds from live bird markets | Ada | Greater Accra | Southern |
| 17 | ADA7 | Rural free-range birds from live bird markets | Ada | Greater Accra | Southern |
| 18 | ADA8 | Rural free-range birds from live bird markets | Ada | Greater Accra | Southern |
| 19 | ADA9 | Rural free-range birds from live bird markets | Ada | Greater Accra | Southern |
| 20 | ADA10 | Rural free-range birds from live bird markets | Ada | Greater Accra | Southern |
| 21 | KAN5 | Urban free-range from communities within Accra | Kaneshie | Greater Accra | Southern |
| 22 | KAN6 | Urban free-range from communities within Accra | Kaneshie | Greater Accra | Southern |
| 23 | WEIJ1 | Urban free-range from communities within Accra | Weija | Greater Accra | Southern |
| 24 | WEIJ2 | Urban free-range from communities within Accra | Weija | Greater Accra | Southern |
| 25 | WEIJ3 | Urban free-range from communities within Accra | Weija | Greater Accra | Southern |
| 26 | WEIJ4 | Urban free-range from communities within Accra | Weija | Greater Accra | Southern |
| 27 | WEIJ5 | Urban free-range from communities within Accra | Weija | Greater Accra | Southern |
| 28 | WEIJ6 | Urban free-range from communities within Accra | Weija | Greater Accra | Southern |
| 29 | WEIJ7 | Urban free-range from communities within Accra | Weija | Greater Accra | Southern |
| 30 | WEIJ8 | Urban free-range from communities within Accra | Weija | Greater Accra | Southern |
| 31 | WEIJ9 | Urban free-range from communities within Accra | Weija | Greater Accra | Southern |
| 32 | WEIJ10 | Urban free-range from communities within Accra | Weija | Greater Accra | Southern |
| 33 | WEIJ11 | Urban free-range from communities within Accra | Weija | Greater Accra | Southern |
| 34 | WEIJ12 | Urban free-range from communities within Accra | Weija | Greater Accra | Southern |
| 35 | WEIJ13 | Urban free-range from communities within Accra | Weija | Greater Accra | Southern |
| 36 | WEIJ14 | Urban free-range from communities within Accra | Weija | Greater Accra | Southern |
| 37 | DOM13 | Urban free-range from communities within Accra | Dome | Greater Accra | Southern |
| 38 | DOM14 | Urban free-range from communities within Accra | Dome | Greater Accra | Southern |
| 39 | DOM15 | Urban free-range from communities within Accra | Dome | Greater Accra | Southern |
| 40 | DOM16 | Urban free-range from communities within Accra | Dome | Greater Accra | Southern |
| 41 | KAN1 | Industrial (Intensive Bred) From Live Bird Markets | Kaneshie | Greater Accra | Southern |
| 42 | KAN2 | Industrial (Intensive Bred) From Live Bird Markets | Kaneshie | Greater Accra | Southern |
| 43 | KAN3 | Industrial (Intensive Bred) From Live Bird Markets | Kaneshie | Greater Accra | Southern |
| 44 | KAN4 | Industrial (Intensive Bred) From Live Bird Markets | Kaneshie | Greater Accra | Southern |
| 45 | KAN7 | Industrial (Intensive Bred) From Live Bird Markets | Kaneshie | Greater Accra | Southern |
| 46 | KAN8 | Industrial (Intensive Bred) From Live Bird Markets | Kaneshie | Greater Accra | Southern |
| 47 | KAN9 | Industrial (Intensive Bred) From Live Bird Markets | Kaneshie | Greater Accra | Southern |
| 48 | ADA11 | Industrial (Intensive Bred) From Live Bird Markets | Ada | Greater Accra | Southern |
| 49 | DOM1 | Industrial (Intensive Bred) From Live Bird Markets | Dome | Greater Accra | Southern |
| 50 | DOM2 | Industrial (Intensive Bred) From Live Bird Markets | Dome | Greater Accra | Southern |
| 51 | DOM3 | Industrial (Intensive Bred) From Live Bird Markets | Dome | Greater Accra | Southern |
| 52 | DOM4 | Industrial (Intensive Bred) From Live Bird Markets | Dome | Greater Accra | Southern |
| 53 | DOM5 | Industrial (Intensive Bred) From Live Bird Markets | Dome | Greater Accra | Southern |
| 54 | DOM6 | Industrial (Intensive Bred) From Live Bird Markets | Dome | Greater Accra | Southern |
| 55 | DOM7 | Industrial (Intensive Bred) From Live Bird Markets | Dome | Greater Accra | Southern |
| 56 | DOM8 | Industrial (Intensive Bred) From Live Bird Markets | Dome | Greater Accra | Southern |
| 57 | DOM9 | Industrial (Intensive Bred) From Live Bird Markets | Dome | Greater Accra | Southern |
| 58 | DOM10 | Industrial (Intensive Bred) From Live Bird Markets | Dome | Greater Accra | Southern |
| 59 | DOM11 | Industrial (Intensive Bred) From Live Bird Markets | Dome | Greater Accra | Southern |
| 60 | DOM12 | Industrial (Intensive Bred) From Live Bird Markets | Dome | Greater Accra | Southern |

**Table S3**: Summary table of the 90 samples from Ghana containing the total number of fragments per sample (fragmentCount), the total number of fragments per sample aligned to a reference from the Silva database (Silva_fragmentCountAln) also shown as percent of total fragment count (Silva_percentAln), the total number of fragments per sample aligned to a reference from the ResFinder database (ResFinder_fragmentCountAln) also shown as percent of total fragment count (ResFinder_percentAln).

| **Sample** | **fragmentCount** | **Silva_fragmentCountAln** | **Silva_percentAln (%)** | **ResFinder_percentAln (%)** | **ResFinder_fragmentCountAln** |
| --- | --- | --- | --- | --- | --- |
| DTU_2022_1013974_1_MG_GH-EE-MM36_S62_L003_R1_001 | 82894139 | 498657 | 0.6016 | 0.0525 | 43553 |
| DTU_2022_1013975_1_MG_GH-EE-PB61_S0_L001_R1_001 | 43262493 | 243547 | 0.563 | 0.1057 | 45724 |
| DTU_2022_1013986_1_MG_GH-EE-DL54_S63_L003_R1_001 | 77505006 | 482216 | 0.6222 | 0.0583 | 45186 |
| DTU_2022_1014000_1_MG_GH-EE-TO52_S1_L004_R1_001 | 77498776 | 356259 | 0.4597 | 0.0669 | 51879 |
| DTU_2022_1013962_1_MG_GH-VS-DOM3_S6_L004_R1_001 | 55999190 | 150660 | 0.269 | 0.0952 | 53301 |
| DTU_2022_1013982_1_MG_GH-EE-SC34_S76_L003_R1_001 | 53895437 | 135734 | 0.2518 | 0.0595 | 32072 |
| DTU_2022_1013985_1_MG_GH-EE-AK47_S3_L003_R1_001 | 59028947 | 204588 | 0.3466 | 0.1014 | 59853 |
| DTU_2022_1013999_1_MG_GH-EE-EK55_S65_L003_R1_001 | 78944719 | 346771 | 0.4393 | 0.1459 | 115191 |
| DTU_2022_1013988_1_MG_GH-EE-JKY71_S66_L003_R1_001 | 89947816 | 506761 | 0.5634 | 0.064 | 57593 |
| DTU_2022_1013961_1_MG_GH-VS-DOM2_S1_L003_R1_001 | 43699656 | 82215 | 0.1881 | 0.1635 | 71431 |
| DTU_2022_1013938_1_MG_GH-VS-WEIJ5_S177_L004_R1_001 | 92266302 | 174980 | 0.1896 | 0.0265 | 24471 |
| DTU_2022_1013941_1_MG_GH-VS-WEIJ8_S190_L004_R1_001 | 71542024 | 124573 | 0.1741 | 0.0498 | 35638 |
| DTU_2022_1013954_1_MG_GH-VS-KAN3_S21_L002_R1_001 | 75995696 | 145458 | 0.1914 | 0.2344 | 178125 |
| DTU_2022_1014001_1_MG_GH-EE-ST37_S12_L003_R1_001 | 61131014 | 256415 | 0.4195 | 0.0872 | 53282 |
| DTU_2022_1013979_1_MG_GH-EE-ANY42_S71_L003_R1_001 | 75582719 | 237883 | 0.3147 | 0.0911 | 68876 |
| DTU_2022_1013948_1_MG_GH-VS-DOM13_S0_L001_R1_001 | 42521447 | 112781 | 0.2652 | 0.0519 | 22075 |
| DTU_2022_1013956_1_MG_GH-VS-KAN7_S0_L001_R1_001 | 46513507 | 92096 | 0.198 | 0.1925 | 89528 |
| DTU_2022_1013963_1_MG_GH-VS-DOM4_S0_L001_R1_001 | 51117263 | 89444 | 0.175 | 0.2464 | 125957 |
| DTU_2022_1013951_1_MG_GH-VS-DOM16_S182_L004_R1_001 | 46296604 | 90944 | 0.1964 | 0.0676 | 31290 |
| DTU_2022_1013978_1_MG_GH-EE-CON40_S68_L003_R1_001 | 99545585 | 334482 | 0.336 | 0.0539 | 53693 |
| DTU_2022_1013995_1_MG_GH-EE-JY65_S27_L003_R1_001 | 45483472 | 334063 | 0.7345 | 0.0544 | 24737 |
| DTU_2022_1013953_1_MG_GH-VS-KAN2_S191_L004_R1_001 | 43302141 | 95394 | 0.2203 | 0.2229 | 96502 |
| DTU_2022_1013991_1_MG_GH-EE-NND65_S2_L004_R1_001 | 90818811 | 565092 | 0.6222 | 0.0512 | 46485 |
| DTU_2022_1013970_1_MG_GH-VS-DOM11_S48_L003_R1_001 | 47826305 | 91681 | 0.1917 | 0.3052 | 145987 |
| DTU_2022_1013981_1_MG_GH-EE-EOA39_S1_L001_R1_001 | 72127814 | 228290 | 0.3165 | 0.082 | 59152 |
| DTU_2022_1013984_1_MG_GH-EE-VG62_S3_L004_R1_001 | 154286759 | 485003 | 0.3144 | 0.0911 | 140569 |
| DTU_2022_1013920_1_MG_GH-VS-ASH9_S11_L002_R1_001 | 69168414 | 125664 | 0.1817 | 0.1722 | 119139 |
| DTU_2022_1013973_1_MG_GH-EE-CA63_S2_L003_R1_001 | 52450289 | 295379 | 0.5632 | 0.0536 | 28125 |
| DTU_2022_1013990_1_MG_GH-EE-LA74_S69_L003_R1_001 | 86139340 | 451918 | 0.5246 | 0.1134 | 97693 |
| DTU_2022_1013980_1_MG_GH-EE-KF52_S86_L003_R1_001 | 57605208 | 210011 | 0.3646 | 0.0652 | 37541 |
| DTU_2022_1013989_1_MG_GH-EE-FKA76_S85_L003_R1_001 | 70459541 | 200433 | 0.2845 | 0.1112 | 78375 |
| DTU_2022_1013994_1_MG_GH-EE-BB27_S1_L003_R1_001 | 68212405 | 275809 | 0.4043 | 0.0599 | 40889 |
| DTU_2022_1013926_1_MG_GH-VS-ADA5_S176_L004_R1_001 | 57326670 | 91753 | 0.1601 | 0.1195 | 68520 |
| DTU_2022_1013967_1_MG_GH-VS-DOM8_S0_L001_R1_001 | 42358972 | 76282 | 0.1801 | 0.1773 | 75118 |
| DTU_2022_1013971_1_MG_GH-VS-DOM12_S25_L003_R1_001 | 51118910 | 108220 | 0.2117 | 0.0879 | 44950 |
| DTU_2022_1013992_1_MG_GH-EE-EJ37_S0_L001_R1_001 | 42838608 | 201978 | 0.4715 | 0.1888 | 80897 |
| DTU_2022_1013933_1_MG_GH-VS-KAN6_S13_L002_R1_001 | 104298000 | 200697 | 0.1924 | 0.0243 | 25316 |
| DTU_2022_1013931_1_MG_GH-VS-ADA10_S3_L002_R1_001 | 103959320 | 213786 | 0.2056 | 0.1927 | 200380 |
| DTU_2022_1013987_1_MG_GH-EE-BAK63_S6_L003_R1_001 | 52319553 | 144433 | 0.2761 | 0.0655 | 34267 |
| DTU_2022_1013959_1_MG_GH-VS-ADA11_S209_L004_R1_001 | 66080361 | 137165 | 0.2076 | 0.2901 | 191696 |
| DTU_2022_1013952_1_MG_GH-VS-KAN1_S32_L001_R1_001 | 55703245 | 116098 | 0.2084 | 0.2157 | 120139 |
| DTU_2022_1013915_1_MG_GH-VS-ASH4_S179_L004_R1_001 | 60302556 | 149751 | 0.2483 | 0.2432 | 146651 |
| DTU_2022_1013968_1_MG_GH-VS-DOM9_S80_L003_R1_001 | 42799005 | 87003 | 0.2033 | 0.1489 | 63715 |
| DTU_2022_1013969_1_MG_GH-VS-DOM10_S0_L001_R1_001 | 42390677 | 95780 | 0.2259 | 0.2051 | 86954 |
| DTU_2022_1013912_1_MG_GH-VS-ASH1_S24_L001_R1_001 | 57438643 | 110604 | 0.1926 | 0.0752 | 43219 |
| DTU_2022_1013966_1_MG_GH-VS-DOM7_S15_L003_R1_001 | 57593423 | 110337 | 0.1916 | 0.183 | 105398 |
| DTU_2022_1013997_1_MG_GH-EE-SAY52_S61_L003_R1_001 | 95908031 | 354120 | 0.3692 | 0.1415 | 135709 |
| DTU_2022_1013927_1_MG_GH-VS-ADA6_S180_L004_R1_001 | 47548977 | 91834 | 0.1931 | 0.1124 | 53442 |
| DTU_2022_1013958_1_MG_GH-VS-KAN9_S29_L002_R1_001 | 71101477 | 146534 | 0.2061 | 0.2511 | 178504 |
| DTU_2022_1013939_1_MG_GH-VS-WEIJ6_S181_L004_R1_001 | 45091727 | 83691 | 0.1856 | 0.0257 | 11576 |
| DTU_2022_1013993_1_MG_GH-EE-SAK32_S0_L001_R1_001 | 47703061 | 274437 | 0.5753 | 0.1309 | 62437 |
| DTU_2022_1013950_1_MG_GH-VS-DOM15_S178_L004_R1_001 | 61446786 | 118999 | 0.1937 | 0.2335 | 143491 |
| DTU_2022_1013960_1_MG_GH-VS-DOM1_S25_L001_R1_001 | 68329790 | 131576 | 0.1926 | 0.2258 | 154296 |
| DTU_2022_1013934_1_MG_GH-VS-WEIJ1_S15_L002_R1_001 | 15263247 | 63379 | 0.4152 | 0.0154 | 2348 |
| DTU_2022_1013935_1_MG_GH-VS-WEIJ2_S207_L004_R1_001 | 51310273 | 79038 | 0.154 | 0.0167 | 8565 |
| DTU_2022_1013965_1_MG_GH-VS-DOM6_S0_L001_R1_001 | 43439001 | 80702 | 0.1858 | 0.2404 | 104424 |
| DTU_2022_1013913_1_MG_GH-VS-ASH2_S171_L004_R1_001 | 53402161 | 115409 | 0.2161 | 0.076 | 40572 |
| DTU_2022_1013964_1_MG_GH-VS-DOM5_S8_L003_R1_001 | 69583743 | 120038 | 0.1725 | 0.2653 | 184595 |
| DTU_2022_1013944_1_MG_GH-VS-WEIJ11_S26_L002_R1_001 | 45767494 | 95977 | 0.2097 | 0.2521 | 115381 |
| DTU_2022_1013943_1_MG_GH-VS-WEIJ10_S24_L002_R1_001 | 54231917 | 107657 | 0.1985 | 0.0419 | 22746 |
| DTU_2022_1013929_1_MG_GH-VS-ADA8_S189_L004_R1_001 | 72000804 | 132349 | 0.1838 | 0.1489 | 107181 |
| DTU_2022_1013917_1_MG_GH-VS-ASH6_S188_L004_R1_001 | 57701449 | 112399 | 0.1948 | 0.074 | 42674 |
| DTU_2022_1013998_1_MG_GH-EE-AT52_S64_L003_R1_001 | 82662607 | 329833 | 0.399 | 0.169 | 139734 |
| DTU_2022_1013924_1_MG_GH-VS-ADA3_S31_L002_R1_001 | 70725223 | 201581 | 0.285 | 0.1387 | 98090 |
| DTU_2022_1013921_1_MG_GH-VS-ASH10_S35_L001_R1_001 | 49314499 | 78064 | 0.1583 | 0.0478 | 23596 |
| DTU_2022_1013932_1_MG_GH-VS-KAN5_S12_L002_R1_001 | 59760957 | 86865 | 0.1454 | 0.0186 | 11121 |
| DTU_2022_1013957_1_MG_GH-VS-KAN8_S202_L004_R1_001 | 49759985 | 93731 | 0.1884 | 0.2115 | 105239 |
| DTU_2022_1013949_1_MG_GH-VS-DOM14_S16_L002_R1_001 | 51064169 | 108694 | 0.2129 | 0.0448 | 22901 |
| DTU_2022_1013923_1_MG_GH-VS-ADA2_S0_L001_R1_001 | 46244725 | 107279 | 0.232 | 0.0628 | 29035 |
| DTU_2022_1013972_1_MG_GH-EE-AS54_S75_L004_R1_001 | 53622011 | 340167 | 0.6344 | 0.1535 | 82298 |
| DTU_2022_1013996_1_MG_GH-EE-EAB56_S9_L004_R1_001 | 48926386 | 207596 | 0.4243 | 0.1485 | 72639 |
| DTU_2022_1013947_1_MG_GH-VS-WEIJ14_S208_L004_R1_001 | 49907516 | 138154 | 0.2768 | 0.1442 | 71946 |
| DTU_2022_1013930_1_MG_GH-VS-ADA9_S195_L004_R1_001 | 65820315 | 130972 | 0.199 | 0.1194 | 78566 |
| DTU_2022_1013936_1_MG_GH-VS-WEIJ3_S16_L001_R1_001 | 52611509 | 189993 | 0.3611 | 0.0218 | 11485 |
| DTU_2022_1013922_1_MG_GH-VS-ADA1_S203_L004_R1_001 | 45569003 | 91551 | 0.2009 | 0.0301 | 13739 |
| DTU_2022_1013983_1_MG_GH-EE-KN57_S0_L001_R1_001 | 42997592 | 148399 | 0.3451 | 0.1044 | 44877 |
| DTU_2022_1013918_1_MG_GH-VS-ASH7_S194_L004_R1_001 | 57431759 | 136047 | 0.2369 | 0.0308 | 17699 |
| DTU_2022_1013937_1_MG_GH-VS-WEIJ4_S0_L001_R1_001 | 54711049 | 113948 | 0.2083 | 0.0111 | 6062 |
| DTU_2022_1013977_1_MG_GH-EE-TYT24_S67_L003_R1_001 | 83138217 | 418357 | 0.5032 | 0.0937 | 77937 |
| DTU_2022_1013914_1_MG_GH-VS-ASH3_S175_L004_R1_001 | 67194189 | 120698 | 0.1796 | 0.0269 | 18063 |
| DTU_2022_1013940_1_MG_GH-VS-WEIJ7_S15_L001_R1_001 | 51943723 | 191069 | 0.3678 | 0.0035 | 1824 |
| DTU_2022_1013976_1_MG_GH-EE-JKA41_S9_L003_R1_001 | 44358734 | 186497 | 0.4204 | 0.0725 | 32166 |
| DTU_2022_1013919_1_MG_GH-VS-ASH8_S197_L004_R1_001 | 50220013 | 83982 | 0.1672 | 0.1788 | 89785 |
| DTU_2022_1013945_1_MG_GH-VS-WEIJ12_S201_L004_R1_001 | 50102160 | 112514 | 0.2246 | 0.2398 | 120125 |
| DTU_2022_1013955_1_MG_GH-VS-KAN4_S25_L002_R1_001 | 49457862 | 100527 | 0.2033 | 0.1448 | 71613 |
| DTU_2022_1013942_1_MG_GH-VS-WEIJ9_S10_L002_R1_001 | 52216618 | 171821 | 0.3291 | 0.0205 | 10719 |
| DTU_2022_1013946_1_MG_GH-VS-WEIJ13_S204_L004_R1_001 | 55456178 | 135313 | 0.244 | 0.1966 | 109021 |
| DTU_2022_1013916_1_MG_GH-VS-ASH5_S0_L001_R1_001 | 46186428 | 98843 | 0.214 | 0.0664 | 30660 |
| DTU_2022_1013925_1_MG_GH-VS-ADA4_S172_L004_R1_001 | 63957018 | 115336 | 0.1803 | 0.1564 | 100027 |
| DTU_2022_1013928_1_MG_GH-VS-ADA7_S185_L004_R1_001 | 65993561 | 121488 | 0.1841 | 0.1566 | 103371 |

**Table S4:** Top 10 most abundant bacterial genera in pig and poultry samples from Ghana.

| **Pig samples** | | **Poultry samples** | |
| --- | --- | --- | --- |
| **Bacterial genera** | **clr median** | **Bacterial genera** | **clr median** |
| *Subdoligranulum* | 11.798164 | *Subdoligranulum* | 11.30452 |
| *Streptococcus* | 10.6342 | *Streptococcus* | 8.900365 |
| *Lactobacillus* | 9.698204 | *Olsenella* | 7.821673 |
| *Bifidobacterium* | 8.289015 | *Bacteroides* | 7.76801 |
| *Myroides* | 7.372511 | *Myroides* | 7.625704 |
| *Clostridium* | 7.310672 | *unknown* | 7.556347 |
| *unknown* | 7.29591 | *Enterococcus* | 7.209787 |
| *Romboutsia* | 6.817021 | *Klebsiella* | 6.734416 |
| *Bacillus* | 6.80267 | *Lactobacillus* | 6.609951 |
| *Klebsiella* | 6.777684 | *Parabacteroides* | 6.261142 |

**Table S5**: Top 10 most abundant resistance genes in poultry and pig samples from Ghana.

| **Poultry samples** | | **Pig samples** | |
| --- | --- | --- | --- |
| Resistance gene | Clr median | Resistance gene | Clr median |
| *tet(W)_5_AJ427422* | 8.400 | *tet(W)_5_AJ427422* | 8.348 |
| *tet(Q)_1_L33696* | 8.371 | *ant(6)-Ia_3_KF864551* | 8.002 |
| *aph(3')-III_1_M26832* | 7.957 | *tet(O/W)_4_AM889121* | 7.976 |
| *ant(6)-Ia_3_KF864551* | 7.856 | *tet(40)_1_FJ158002* | 7.413 |
| *erm(F)_3_M17808* | 7.759 | *tet(Q)_1_L33696* | 7.060 |
| *ant(6)-Ia_1_AF330699* | 6.966 | *tet(W)_4_FN396364* | 6.908 |
| *tet(40)_1_FJ158002* | 6.944 | *tet(O/W)_5_AM889122* | 6.681 |
| *tet(O/W)_4_AM889121* | 6.457 | *tet(44)_1_NZ_ABDU01000081* | 6.680 |
| *tet(W)_4_FN396364* | 6.417 | *tet(44)_2_FN594949* | 6.649 |
| *tet(X)_2_M37699* | 6.404 | *ant(6)-Ib_1_FN594949* | 6.153 |

**Table S6:** Differential abundant antimicrobial resistance genes (with FDR correction < 0.05) driving resistome differences between European and Ghanaian pigs.

| **Driving Ghanaian pigs** | **Effect** | **Driving European pigs** | **Effect** |
| --- | --- | --- | --- |
| *tetB.P._1_NC_010937* | 1.741 | *lnu.C._1_AY928180* | -2.099 |
| *tet.A._4_AJ517790* | 1.394 | *nimH_1_KX576455* | -2.006 |
| *tet.Z._1_AF121000* | 1.359 | *nimJ_1_NZ_JH815495* | -1.710 |
| *str_1_X92946* | 1.233 | *tet.Q._2_X58717* | -1.530 |
| *tet.O.W._3_AM889120* | 1.192 | *mef.A._3_AF227520* | -1.438 |
| *mdf.A._1_Y08743* | 1.182 | *blaACI.1_1_AJ007350* | -1.323 |
| *cmx_1_U85507* | 1.120 | *erm.G._1_M15332* | -1.299 |
| *tet.O.W._4_AM889121* | 1.090 | *tet.Q._3_U73497* | -1.260 |
| *str_2_FN435330* | 1.039 | *tet.X._1_GU014535* | -1.247 |
| *tet.O.W._5_AM889122* | 0.967 | *erm.G._2_L42817* | -1.242 |
| *tetA.P._2_L20800* | 0.945 | *tet.X._2_M37699* | -1.241 |
| *tetA.P._1_AB054980* | 0.904 | *tet.Q._1_L33696* | -1.231 |
| *cat_5_U35036* | 0.871 | *mef.A._2_U83667* | -1.228 |
| *sul1_5_EU780013* | 0.807 | *cfr.C._2_CANB01000378* | -1.205 |
| *qnrB19_1_EU432277* | 0.764 | *tet.Q._4_Z21523* | -1.202 |
| *sul2_2_AY034138* | 0.744 | *mef.A._1_AJ971089* | -1.141 |
| *tet.33._1_AY255627* | 0.731 | *cfxA6_1_GQ342996* | -1.135 |
| *aph.3....Ib_5_AF321551* | 0.711 | *VanG2XY_1_FJ872410* | -1.077 |
| *aadA9_1_AJ420072* | 0.688 | *mef.A._4_HG423652* | -1.023 |
| *tet.A._6_AF534183* | 0.682 | *erm.F._3_M17808* | -0.992 |
| *aac.6...Ii_1_L12710* | 0.680 | *erm.F._4_M62487* | -0.980 |
| *tet.44._1_NZ_ABDU01000081* | 0.669 | *nimB_1_X71443* | -0.912 |
| *tet.33._2_DQ390458* | 0.648 | *cfxA_1_U38243* | -0.812 |
| *dfrG_1_AB205645* | 0.644 | *cfxA2_1_AF504914* | -0.771 |
| *msr.C._2_AF313494* | 0.640 | *VanGXY_1_AY271782* | -0.763 |
| *tet.M._7_FN433596* | 0.600 | *VanHBX_1_AF192329* | -0.737 |
| *tet.L._1_HM235948* | 0.577 | *erm.B._12_U18931* | -0.693 |
| *tet.W._4_FN396364* | 0.565 | *mph.B._1_D85892* | -0.553 |
| *aph.6..Id_1_M28829* | 0.546 | *aph.2....Ib_2_AF207840* | -0.552 |
| *tet.M._2_X90939* | 0.521 | *nimC_1_X76948* | -0.550 |
| *aadA2_1_NC_010870* | 0.520 | *lsa.C._1_HM990671* | -0.549 |
| *erm.X._4_NC_005206* | 0.515 | *cfxA3_1_AF472622* | -0.523 |
| *aph.3....Ib_2_AF024602* | 0.514 | *mph.N._1_KF648874* | -0.513 |
| *tet.S.M._2_AY534326* | 0.511 | *lnu.P._1_FJ589781* | -0.502 |
| *tet.M._13_AM990992* | 0.494 | *cfr.C._1_KX686749* | -0.490 |
| *tet.O.W._2_AM889119* | 0.478 | *ant.9..Ia_1_X02588* | -0.464 |
| *tet.O.32.O._2_AJ295238* | 0.470 | *lnu.A._1_M14039* | -0.452 |
| *tet.M._12_FR671418* | 0.453 | *aph.2....Ih_1_KF652096* | -0.426 |
| *ant.6..Ia_2_KF421157* | 0.378 | *erm.A._3_EU348758* | -0.414 |
| *tet.39._1_KT346360* | 0.332 | *erm.33._1_AJ579365* | -0.409 |
|  |  | *aac.6...Im_1_AF337947* | -0.406 |
|  |  | *ant.6..Ia_1_AF330699* | -0.388 |
|  |  | *tet.36._1_AJ514254* | -0.382 |

**Table S7:** Differential abundant antimicrobial resistance genes (with FDR correction < 0.05) driving resistome differences between European and Ghanaian poultry.

| **Driving Ghanaian poultry** | **Effect** | **Driving European poultry** | **Effect** |
| --- | --- | --- | --- |
| *tet.W._5_AJ427422* | 2.184 | *lnu.A._1_M14039* | -2.558 |
| *tet.Q._1_L33696* | 1.627 | *blaTEM.1B_1_AY458016* | -1.724 |
| *tet.Q._3_U73497* | 1.589 | *ant.3....Ia_1_X02340* | -1.711 |
| *tet.40._1_FJ158002* | 1.463 | *dfrD_1_Z50141* | -1.543 |
| *tet.O._2_M20925* | 1.451 | *dfrA1_5_EU089668* | -1.448 |
| *tet.40._2_AM419751* | 1.439 | *mph.C._2_AF167161* | -1.412 |
| *tet.O._3_Y07780* | 1.412 | *ant.9..Ia_1_X02588* | -1.373 |
| *tet.Q._2_X58717* | 1.338 | *mdf.A._1_Y08743* | -1.359 |
| *tet.X._3_AB097942* | 1.265 | *tet.A._6_AF534183* | -1.333 |
| *tet.O.32.O._2_AJ295238* | 1.219 | *tet.33._1_AY255627* | -1.316 |
| *tet.O.32.O._5_FP929050* | 1.210 | *aadA6_1_AF140629* | -1.290 |
| *erm.F._3_M17808* | 1.201 | *dfrA1_8_X00926* | -1.263 |
| *ant.6..Ia_3_KF864551* | 1.120 | *tet.K._1_U38656* | -1.244 |
| *nimB_1_X71443* | 1.117 | *aadA2_1_NC_010870* | -1.238 |
| *tet.W._2_AY049983* | 1.106 | *msr.A._2_AB013298* | -1.208 |
| *tet.X._1_GU014535* | 1.092 | *sul1_9_AY963803* | -1.204 |
| *tet.W._1_DQ060146* | 1.078 | *dfrA1_10_AF203818* | -1.091 |
| *tet.X._2_M37699* | 1.045 | *sul1_5_EU780013* | -1.082 |
| *blaOXA.347_1_ACWG01000053* | 1.004 | *lnu.G._1_KX470419* | -1.063 |
| *sul2_18_AJ830714* | 0.943 | *vat.E._5_AJ488494* | -1.052 |
| *tet.X4._1_MK134376* | 0.872 | *msr.A._1_X52085* | -1.039 |
| *cfxA2_1_AF504914* | 0.852 | *dfrK_1_FN377602* | -1.014 |
| *mef.A._3_AF227520* | 0.839 | *tet.B._2_AF326777* | -1.013 |
| *erm.F._4_M62487* | 0.837 | *cmx_1_U85507* | -0.998 |
| *tet.O.32.O._1_JQ740052* | 0.818 | *tet.33._2_DQ390458* | -0.998 |
| *mef.A._1_AJ971089* | 0.783 | *tet.Z._1_AF121000* | -0.974 |
| *tet.32._2_EF626943* | 0.776 | *mph.C._1_AB013298* | -0.968 |
| *cfxA_1_U38243* | 0.775 | *lnu.P._1_FJ589781* | -0.958 |
| *cfxA6_1_GQ342996* | 0.746 | *dfrA15_2_AF221900* | -0.952 |
| *mef.A._2_U83667* | 0.735 | *mph.B._1_D85892* | -0.930 |
| *mef.A._4_HG423652* | 0.711 | *erm.B._15_U48430* | -0.923 |
| *tet.Q._4_Z21523* | 0.702 | *erm.B._6_AF242872* | -0.923 |
| *tet.O.32.O._4_AIOQ01000025* | 0.670 | *blaVIM.48_1_KY362199* | -0.914 |
| *tet.O.W._3_AM889120* | 0.667 | *mph.C._3_AM180068* | -0.897 |
| *cfxA3_1_AF472622* | 0.653 | *aac.6...Im_1_AF337947* | -0.891 |
| *nimC_1_X76948* | 0.616 | *lnu.C._1_AY928180* | -0.881 |
| *ant.6..Ia_1_AF330699* | 0.606 | *erm.X._4_NC_005206* | -0.866 |
| *tet.X3._1_MK134375* | 0.601 | *poxtA_1_MF095097* | -0.860 |
| *tet.W.32.O._3_AM710603* | 0.578 | *cmlA1_1_M64556* | -0.855 |
| *cfxA5_1_AY769934* | 0.573 | *erm.36._1_AF462611* | -0.836 |
| *tet.32._1_EU722333* | 0.553 | *aadA1_4_JQ480156* | -0.830 |
| *tet.W.32.O._1_AM710601* | 0.550 | *aadA1_5_JX185132* | -0.820 |
| *tet.44._1_NZ_ABDU01000081* | 0.544 | *dfrG_1_AB205645* | -0.817 |
| *tet.O.W..1_1_AY485126* | 0.541 | *erm.43._1_HE650138* | -0.815 |
| *tet.O.32.O._3_NZ_AUJS01000017* | 0.526 | *catA1_1_V00622* | -0.801 |
| *tet.O.W.O..3_1_EF065524* | 0.519 | *mph.C._4_AM397632* | -0.768 |
| *cfr.C._1_KX686749* | 0.514 | *lsa.E._1_JX560992* | -0.766 |
| *aph.3...VIIa_1_M29953* | 0.492 | *lnu.B._2_JQ861959* | -0.747 |
| *tva.A._1_ENA_SOX29786* | 0.478 | *aph.6..Id_1_M28829* | -0.745 |
| *erm.G._2_L42817* | 0.452 | *sul2_2_AY034138* | -0.739 |
| *blaTEM.207_1_KC818234* | 0.367 | *sul3_2_AJ459418* | -0.735 |
| *aph.3...IIIa_3_AB247327* | 0.353 | *aph.2....Ia_2_AP009486* | -0.721 |
| *tet.O.W.32.O._5_JQ740053* | 0.342 | *aph.3....Ib_2_AF024602* | -0.712 |
| *tet.O.W.32.O._1_EF065523* | 0.342 | *blaTEM.1C_1_FJ560503* | -0.711 |
| *aadA8b_2_AM040708* | 0.339 | *fosD_1_KC989517* | -0.679 |
| *aph.3...IIIa_1_AF330699* | 0.337 | *aph.4..Ia_1_V01499* | -0.675 |
| *blaTEM.214_1_KP050491* | 0.331 | *tet.M._13_AM990992* | -0.673 |
| *cfr.C._2_CANB01000378* | 0.322 | *aadA1_3_JQ414041* | -0.666 |
| *dfrA1_2_HM055363* | 0.319 | *mph.C._5_AM180067* | -0.659 |
| *msr.D._3_AF227520* | 0.317 | *mef.B._1_FJ196385* | -0.648 |
| *nimA_1_X71444* | 0.316 | *aadA5_1_AF137361* | -0.638 |
| *blaOXA.85_1_JANA01000064* | 0.297 | *VanHBX_1_AF192329* | -0.637 |
| *msr.D._2_AF274302* | 0.297 | *cml_1_M22614* | -0.618 |
| *tet.K._2_J01764* | 0.290 | *tet.L._8_AY081910* | -0.602 |
| *tet.W.32.O._2_AM710602* | 0.231 | *aadA17_1_FJ460181* | -0.587 |
|  |  | *erm.A._1_X03216* | -0.562 |
|  |  | *aadD_2_M19465* | -0.560 |
|  |  | *erm.C._12_Y09003* | -0.558 |
|  |  | *erm.B._1_JN899585* | -0.549 |
|  |  | *aadD_1_AF181950* | -0.538 |
|  |  | *erm.B._18_X66468* | -0.528 |
|  |  | *sul2_3_HQ840942* | -0.520 |
|  |  | *ant.6..Ia_2_KF421157* | -0.516 |
|  |  | *sul2_6_FN995456* | -0.480 |
|  |  | *erm.T._2_AY894138* | -0.479 |
|  |  | *qnrB19_1_EU432277* | -0.478 |
|  |  | *aph.2....Ib_2_AF207840* | -0.477 |
|  |  | *tet.M._7_FN433596* | -0.471 |
|  |  | *erm.C._13_M13761* | -0.465 |
|  |  | *erm.A._3_EU348758* | -0.445 |
|  |  | *cat.pC233._1_AY355285* | -0.445 |
|  |  | *aph.3....Ib_5_AF321551* | -0.437 |
|  |  | *aadA10_2_AM087405* | -0.423 |
|  |  | *aac.3..IV_1_DQ241380* | -0.422 |
|  |  | *tet.C._3_AF055345* | -0.415 |
|  |  | *str_1_X92946* | -0.413 |
|  |  | *erm.X._1_M36726* | -0.407 |
|  |  | *floR_2_AF118107* | -0.403 |
|  |  | *tet.M._4_X75073* | -0.399 |
|  |  | *erm.33._1_AJ579365* | -0.395 |
|  |  | *erm.Q._1_L22689* | -0.395 |
|  |  | *tet.O.W._4_AM889121* | -0.387 |
|  |  | *tet.L._1_HM235948* | -0.382 |
|  |  | *aac.3..IVa_1_X01385* | -0.379 |
|  |  | *aph.2....Id_1_AF016483* | -0.378 |
|  |  | *aadA9_1_AJ420072* | -0.374 |
|  |  | *erm.B._26_AF080450* | -0.359 |
|  |  | *erm.B._10_U86375* | -0.356 |
|  |  | *mph.N._1_KF648874* | -0.355 |
|  |  | *aadA2_2_JQ364967* | -0.349 |
|  |  | *VanG2XY_1_FJ872410* | -0.346 |
|  |  | *tet.A._4_AJ517790* | -0.342 |
|  |  | *aph.2....Ib_1_AF337947* | -0.335 |
|  |  | *tet.S.M._1_HM367711* | -0.332 |
|  |  | *aadA24_1_AM711129* | -0.331 |
|  |  | *tetA.P._2_L20800* | -0.330 |
|  |  | *dfrA17_9_FJ807902* | -0.327 |
|  |  | *dfrA1_9_AJ238350* | -0.327 |
|  |  | *aph.3....Ib_4_AF313472* | -0.316 |
|  |  | *tet.C._2_AY046276* | -0.314 |
|  |  | *vga.E._1_FR772051* | -0.314 |
|  |  | *erm.X._2_X51472* | -0.314 |
|  |  | *aac.6...Iih_1_AJ584701* | -0.311 |
|  |  | *aadA13_1_AY713504* | -0.311 |
|  |  | *erm.T._4_AJ488494* | -0.310 |
|  |  | *sul2_9_FJ197818* | -0.308 |
|  |  | *aph.3...Ia_1_V00359* | -0.298 |
|  |  | *blaZ_78_KU607301* | -0.295 |
|  |  | *tetB.P._1_NC_010937* | -0.290 |
|  |  | *tet.M._1_X92947* | -0.289 |
|  |  | *tet.L._2_M29725* | -0.283 |
|  |  | *erm.C._1_V01278* | -0.279 |
|  |  | *blaZ_8_HE993884* | -0.274 |
|  |  | *dfrA14_5_DQ388123* | -0.263 |
|  |  | *cat.pC194._1_NC_002013* | -0.261 |
|  |  | *aph.3...Ia_7_X62115* | -0.260 |
|  |  | *blaTEM.141_1_AY956335* | -0.252 |
|  |  | *dfrA12_8_AM040708* | -0.246 |
|  |  | *aph.2....Ic_1_U51479* | -0.243 |
|  |  | *aadA22_1_AM261837* | -0.217 |
|  |  | *tet.W._4_FN396364* | -0.202 |
|  |  | *dfrA17_1_FJ460238* | -0.189 |

**Table S8:** Differential abundant antimicrobial resistance genes (with FDR correction < 0.05) driving resistome differences between Ghanaian pig and poultry.

| **Driving Ghanaian pig samples** | **Effect** | **Driving Ghanaian poultry samples** | **Effect** |
| --- | --- | --- | --- |
| *cat_3_S48276* | 1.572 | *tetA.P._2_L20800* | -2.121 |
| *cfr.C._2_CANB01000378* | 1.542 | *blaACI.1_1_AJ007350* | -1.780 |
| *tet.X._1_GU014535* | 1.291 | *tetB.P._1_NC_010937* | -1.548 |
| *tet.X._2_M37699* | 1.255 | *str_1_X92946* | -1.331 |
| *erm.F._4_M62487* | 1.151 | *tet.W._2_AY049983* | -1.268 |
| *tet.X._3_AB097942* | 1.141 | *cfxA6_1_GQ342996* | -1.264 |
| *erm.F._3_M17808* | 1.059 | *tetA.P._1_AB054980* | -1.257 |
| *tet.32._2_EF626943* | 1.047 | *lnu.P._1_FJ589781* | -1.190 |
| *tet.Q._3_U73497* | 0.991 | *tet.L._1_HM235948* | -1.076 |
| *tet.O.W..1_1_AY485126* | 0.940 | *ant.6..Ia_2_KF421157* | -1.000 |
| *blaOXA.347_1_ACWG01000053* | 0.906 | *tet.O._3_Y07780* | -0.968 |
| *sul2_18_AJ830714* | 0.877 | *tet.O._1_M18896* | -0.938 |
| *aph.2....Ig_1_CP004067* | 0.860 | *erm.Q._1_L22689* | -0.936 |
| *tet.W._1_DQ060146* | 0.812 | *tet.Z._1_AF121000* | -0.918 |
| *tet.40._2_AM419751* | 0.782 | *cat_2_M35190* | -0.879 |
| *tet.X4._1_MK134376* | 0.773 | *tet.O.W._3_AM889120* | -0.793 |
| *tet.O.32.O._2_AJ295238* | 0.750 | *cmx_1_U85507* | -0.787 |
| *tet.Q._1_L33696* | 0.690 | *tet.C._2_AY046276* | -0.778 |
| *tet.32._1_EU722333* | 0.621 | *str_2_FN435330* | -0.696 |
| *tet.W._5_AJ427422* | 0.584 | *tet.33._1_AY255627* | -0.673 |
| *nimC_1_X76948* | 0.574 | *tet.M._7_FN433596* | -0.669 |
| *ant.6..Ia_1_AF330699* | 0.561 | *tet.O.W..2_1_AY485122* | -0.661 |
| *erm.B._12_U18931* | 0.558 | *tet.39._1_KT346360* | -0.650 |
| *tet.M._10_EU182585* | 0.495 | *tet.S.M._2_AY534326* | -0.650 |
|  |  | *mph.N._1_KF648874* | -0.642 |
|  |  | *tet.33._2_DQ390458* | -0.631 |
|  |  | *catQ_1_M55620* | -0.616 |
|  |  | *tet.O.W.32.O._5_JQ740053* | -0.604 |
|  |  | *aadA9_1_AJ420072* | -0.569 |
|  |  | *tet.44._1_NZ_ABDU01000081* | -0.567 |
|  |  | *tet.O.W._4_AM889121* | -0.559 |
|  |  | *tet.44._2_FN594949* | -0.547 |
|  |  | *cat_5_U35036* | -0.529 |
|  |  | *poxtA_1_MF095097* | -0.515 |
|  |  | *lsa.E._1_JX560992* | -0.500 |
|  |  | *floR_2_AF118107* | -0.499 |
|  |  | *mdf.A._1_Y08743* | -0.493 |
|  |  | *tet.Q._4_Z21523* | -0.487 |
|  |  | *lnu.B._2_JQ861959* | -0.475 |
|  |  | *tet.M._13_AM990992* | -0.463 |
|  |  | *tet.A._6_AF534183* | -0.460 |
|  |  | *tet.O.W._5_AM889122* | -0.459 |
|  |  | *tet.O._2_M20925* | -0.416 |
|  |  | *lnu.C._1_AY928180* | -0.373 |
|  |  | *sul1_5_EU780013* | -0.366 |
|  |  | *ant.6..Ib_1_FN594949* | -0.353 |

**Table S9**: Differential abundant bacterial genera (with FDR correction < 0.05) driving taxa differences between pig and poultry from Ghana.

| **Driving Ghanaian poultry** | **Effect** | **Driving Ghanaian pigs** | **Effect** |
| --- | --- | --- | --- |
| *Alistipes* | 2.566 | *Streptococcus* | -2.283 |
| *Mordavella* | 2.237 | *Paeniclostridium* | -2.162 |
| *Bacteroides* | 2.133 | *Clostridioides* | -2.047 |
| *Enorma* | 1.983 | *Lactobacillus* | -1.955 |
| *Faecalicoccus* | 1.899 | *Romboutsia* | -1.822 |
| *Gorbachella* | 1.784 | *Bifidobacterium* | -1.671 |
| *Mediterranea* | 1.743 | *Roseburia* | -1.605 |
| *Anaerostipes* | 1.702 | *Treponema* | -1.601 |
| *Agathobaculum* | 1.571 | *Weissella* | -1.583 |
| *Faecalitalea* | 1.523 | *Rummeliibacillus* | -1.571 |
| *Massilimicrobiota* | 1.515 | *Nocardia* | -1.568 |
| *Anaerofilum* | 1.509 | *Clostridium* | -1.558 |
| *Flavonifractor* | 1.451 | *Fibrobacter* | -1.546 |
| *Odoribacter* | 1.438 | *Intestinibacter* | -1.499 |
| *Faecalicatena* | 1.330 | *Microcoleus* | -1.357 |
| *Atopobium* | 1.299 | *Kurthia* | -1.348 |
| *Intestinibacillus* | 1.286 | *Corynebacterium* | -1.328 |
| *Barnesiella* | 1.269 | *Candidatus.Phytoplasma* | -1.295 |
| *Peptostreptococcus* | 1.174 | *Mogibacterium* | -1.250 |
| *Intestinimonas* | 1.021 | *Exiguobacterium* | -1.235 |
| *Culturomica* | 1.002 | *Aerococcus* | -1.229 |
| *Ruthenibacterium* | 0.993 | *Galactobacillus* | -1.227 |
| *Polaribacter* | 0.988 | *Staphylococcus* | -1.199 |
| *Sphaerochaeta* | 0.987 | *Anaerovibrio* | -1.151 |
| *Coriobacterium* | 0.969 | *Dubosiella* | -1.135 |
| *Parabacteroides* | 0.951 | *Sinorhizobium* | -1.123 |
| *Porphyromonas* | 0.922 | *Terrisporobacter* | -1.099 |
| *Peptococcus* | 0.920 | *Lachnobacterium* | -1.095 |
| *Olsenella* | 0.919 | *Anaerobium* | -1.081 |
| *Nonlabens* | 0.882 | *Holdemanella* | -1.057 |
| *Faecalibacterium* | 0.865 | *Acinetobacter* | -1.048 |
| *Butyricimonas* | 0.845 | *Acidipropionibacterium* | -1.046 |
| *Catenibacillus* | 0.828 | *Turicibacter* | -1.031 |
| *Mucispirillum* | 0.819 | *Acetivibrio* | -1.020 |
| *Collinsella* | 0.801 | *Lysinibacillus* | -0.977 |
| *Anaerotruncus* | 0.784 | *Mitsuokella* | -0.953 |
| *Paraprevotella* | 0.758 | *Selenomonas* | -0.879 |
| *Tannerella* | 0.752 | *Francisella* | -0.853 |
| *Williamwhitmania* | 0.752 | *Megasphaera* | -0.853 |
| *Bergeyella* | 0.720 | *Viridibacillus* | -0.826 |
| *Spirosoma* | 0.706 | *Anaerocolumna* | -0.824 |
| *Sanguibacteroides* | 0.665 | *Lactococcus* | -0.821 |
| *Enterococcus* | 0.661 | *Anaerolactibacter* | -0.774 |
| *Parasutterella* | 0.661 | *Bacillus* | -0.764 |
| *Halomonas* | 0.652 | *Butyrivibrio* | -0.762 |
| *Candidatus.Stoquefichus* | 0.644 | *Lachnospira* | -0.730 |
| *Lawsonibacter* | 0.627 | *Subdoligranulum* | -0.708 |
| *Phocaeicola* | 0.603 | *Pseudobutyrivibrio* | -0.702 |
| *Coprobacter* | 0.583 | *Listeria* | -0.696 |
| *Veillonella* | 0.551 | *Cellulosilyticum* | -0.649 |
| *Sporobacter* | 0.546 | *Candidatus.Symbiothrix* | -0.638 |
| *Lachnoclostridium* | 0.535 | *Pediococcus* | -0.636 |
| *Paludibacter* | 0.532 | *Dietzia* | -0.631 |
| *Negativibacillus* | 0.517 | *Catenibacterium* | -0.620 |
| *Herbaspirillum* | 0.516 | *Cyanobium* | -0.617 |
| *Marseilla* | 0.509 | *Arsenophonus* | -0.615 |
| *Anaerorhabdus* | 0.494 | *Sporosarcina* | -0.614 |
| *Christensenella* | 0.485 | *Candidatus.Kinetoplastibacterium* | -0.605 |
| *Enterorhabdus* | 0.477 | *Pseudanabaena* | -0.604 |
| *Butyricicoccus* | 0.469 | *Micrococcus* | -0.591 |
| *Aeriscardovia* | 0.462 | *Chlamydia* | -0.588 |
| *Enterobacter* | 0.459 | *Raoultibacter* | -0.579 |
| *Merdibacter* | 0.453 | *Robinsoniella* | -0.579 |
| *Acetobacteroides* | 0.446 | *Epulopiscium* | -0.577 |
| *Fusobacterium* | 0.419 | *Leuconostoc* | -0.576 |
| *Paenibacillus* | 0.281 | *Peptoniphilus* | -0.569 |
|  |  | *Akkermansia* | -0.567 |
|  |  | *Paraclostridium* | -0.556 |
|  |  | *Arthrobacter* | -0.551 |
|  |  | *Serratia* | -0.548 |
|  |  | *Mobilibacterium* | -0.545 |
|  |  | *Escherichia* | -0.537 |
|  |  | *Sharpea* | -0.536 |
|  |  | *Vibrio* | -0.533 |
|  |  | *Merismopedia* | -0.525 |
|  |  | *Salmonella* | -0.525 |
|  |  | *Chishuiella* | -0.525 |
|  |  | *Shigella* | -0.520 |
|  |  | *Parolsenella* | -0.519 |
|  |  | *Hormoscilla* | -0.509 |
|  |  | *Erwinia* | -0.504 |
|  |  | *Parascardovia* | -0.497 |
|  |  | *Synechococcus* | -0.477 |
|  |  | *Nodosilinea* | -0.461 |
|  |  | *Halobacillus* | -0.440 |
|  |  | *Lactimicrobium* | -0.440 |
|  |  | *Campylobacter* | -0.424 |
|  |  | *Oculatella* | -0.412 |
|  |  | *Microbacterium* | -0.395 |
|  |  | *Anaeromassilibacillus* | -0.394 |

**Table S10:** Differential abundant bacterial genera (with FDR correction < 0.05) driving taxa differences between poultry from Ghana and Europe.

| **Driving Ghanaian poultry** | **Effect** | **Driving European poultry** | **Effect** |
| --- | --- | --- | --- |
| *Subdoligranulum* | 6.499 | *Lactobacillus* | -2.695 |
| *Olsenella* | 2.665 | *Serratia* | -2.481 |
| *Collinsella* | 2.627 | *Streptococcus* | -2.355 |
| *Enorma* | 2.352 | *Francisella* | -1.957 |
| *Mediterranea* | 1.981 | *Arsenophonus* | -1.854 |
| *Halomonas* | 1.971 | *Salmonella* | -1.843 |
| *Mordavella* | 1.909 | *Escherichia* | -1.832 |
| *Faecalicoccus* | 1.844 | *Erwinia* | -1.796 |
| *Atopobium* | 1.687 | *Candidatus.Arthromitus* | -1.711 |
| *Sphaerochaeta* | 1.574 | *Shigella* | -1.683 |
| *Tannerella* | 1.556 | *Brachybacterium* | -1.635 |
| *Slackia* | 1.542 | *Staphylococcus* | -1.628 |
| *Prevotella* | 1.492 | *Corynebacterium* | -1.621 |
| *Paenibacillus* | 1.439 | *Jeotgalicoccus* | -1.612 |
| *Porphyromonas* | 1.420 | *Brevibacterium* | -1.585 |
| *Paraprevotella* | 1.303 | *Dickeya* | -1.437 |
| *Muribaculum* | 1.299 | *Klebsiella* | -1.417 |
| *Faecalitalea* | 1.291 | *Vibrio* | -1.363 |
| *Anoxybacillus* | 1.267 | *Weissella* | -1.318 |
| *Polaribacter* | 1.248 | *Romboutsia* | -1.271 |
| *Coriobacterium* | 1.218 | *Microcoleus* | -1.070 |
| *Photobacterium* | 1.213 | *Pseudomonas* | -1.065 |
| *Peptostreptococcus* | 1.176 | *Aerococcus* | -0.908 |
| *Dorea* | 1.167 | *Pectobacterium* | -0.907 |
| *Gorbachella* | 1.141 | *Candidatus.Kinetoplastibacterium* | -0.901 |
| *Bergeyella* | 1.140 | *Rubneribacter* | -0.877 |
| *Treponema* | 1.134 | *Faecalibacterium* | -0.859 |
| *Spirochaeta* | 1.019 | *Cronobacter* | -0.848 |
| *Enterorhabdus* | 1.010 | *Morganella* | -0.847 |
| *Nodosilinea* | 0.996 | *Myroides* | -0.814 |
| *Denitrobacterium* | 0.994 | *Pseudanabaena* | -0.805 |
| *Christensenella* | 0.985 | *Pantoea* | -0.804 |
| *Parabacteroides* | 0.980 | *Proteus* | -0.739 |
| *Candidatus.Stoquefichus* | 0.867 | *Citrobacter* | -0.728 |
| *Barnesiella* | 0.862 | *Gallibacterium* | -0.723 |
| *Mucispirillum* | 0.861 | *Bordetella* | -0.715 |
| *Culturomica* | 0.854 | *Terrisporobacter* | -0.702 |
| *Massilimicrobiota* | 0.835 | *Oceanobacillus* | -0.687 |
| *Faecalicatena* | 0.834 | *Butyricicoccus* | -0.632 |
| *Dysgonomonas* | 0.814 | *Nosocomiicoccus* | -0.581 |
| *Odoribacter* | 0.806 | *Prauserella* | -0.553 |
| *Intestinimonas* | 0.789 | *Candidatus.Phytoplasma* | -0.552 |
| *Erysipelatoclostridium* | 0.787 | *Acinetobacter* | -0.540 |
| *Aeriscardovia* | 0.786 | *Shewanella* | -0.539 |
| *Williamwhitmania* | 0.766 | *Enteractinococcus* | -0.539 |
| *Peptococcus* | 0.759 | *Bacillus* | -0.522 |
| *Erysipelothrix* | 0.755 | *Virgibacillus* | -0.514 |
| *Ruthenibacterium* | 0.749 | *Halobacillus* | -0.503 |
| *Anaerostipes* | 0.746 | *Clostridioides* | -0.503 |
| *Acidithiobacillus* | 0.743 | *Dietzia* | -0.501 |
| *Paludibacter* | 0.733 | *Oenococcus* | -0.489 |
| *Nonlabens* | 0.718 | *Campylobacter* | -0.480 |
| *Eggerthella* | 0.707 | *Gordonia* | -0.476 |
| *Alloprevotella* | 0.686 | *Salinicoccus* | -0.475 |
| *Cyanobium* | 0.669 | *Sediminibacillus* | -0.467 |
| *Brachyspira* | 0.664 | *Sporosarcina* | -0.460 |
| *Leifsonia* | 0.660 | *Globicatella* | -0.448 |
| *Blautia* | 0.658 | *Mycobacterium* | -0.433 |
| *Bacteroides* | 0.648 | *Oculatella* | -0.422 |
| *Coprobacter* | 0.636 | *Atopostipes* | -0.406 |
| *Desulfovibrio* | 0.627 | *Dehalobacter* | -0.400 |
| *Lactimicrobium* | 0.623 | *Janibacter* | -0.399 |
| *Clostridium* | 0.618 | *Facklamia* | -0.370 |
| *Marseilla* | 0.604 | *Rothia* | -0.360 |
| *Gordonibacter* | 0.599 | *Nocardiopsis* | -0.357 |
| *Anaerofilum* | 0.594 | *Eisenbergiella* | -0.343 |
| *Acholeplasma* | 0.576 | *Caedibacter* | -0.331 |
| *Fusobacterium* | 0.576 | *Ureaplasma* | -0.320 |
| *Flavobacterium* | 0.574 | *Marinilactibacillus* | -0.320 |
| *Flavonifractor* | 0.566 | *Kribbella* | -0.318 |
| *Tenacibaculum* | 0.559 | *Rathayibacter* | -0.316 |
| *Lachnoclostridium* | 0.552 | *Trichococcus* | -0.303 |
| *Turicibacter* | 0.550 | *Carnobacterium* | -0.303 |
| *Paraeggerthella* | 0.549 | *Amphibacillus* | -0.303 |
| *Roseburia* | 0.543 | *Bilophila* | -0.302 |
| *Herbaspirillum* | 0.537 | *Alcaligenes* | -0.302 |
| *Anaerorhabdus* | 0.524 | *Micrococcus* | -0.288 |
| *Traorella* | 0.523 | *Paenalcaligenes* | -0.284 |
| *Sanguibacteroides* | 0.521 | *Kurthia* | -0.284 |
| *Spirosoma* | 0.518 | *Paeniclostridium* | -0.283 |
| *Kandleria* | 0.517 | *Megamonas* | -0.277 |
| *Adlercreutzia* | 0.511 | *Oligella* | -0.271 |
| *Marispirochaeta* | 0.504 |  |  |
| *Macellibacteroides* | 0.486 |  |  |
| *Phocaeicola* | 0.475 |  |  |
| *Holdemania* | 0.456 |  |  |
| *Thermotoga* | 0.453 |  |  |
| *Mycoplasma* | 0.448 |  |  |
| *Ruminococcus* | 0.448 |  |  |
| *Sporobacter* | 0.442 |  |  |
| *Cloacibacillus* | 0.425 |  |  |
| *Agathobaculum* | 0.419 |  |  |
| *Candidatus.Armantifilum* | 0.407 |  |  |
| *Borrelia* | 0.384 |  |  |
| *Veillonella* | 0.382 |  |  |
| *Entomoplasma* | 0.380 |  |  |
| *Symbiobacterium* | 0.378 |  |  |
| *Dechloromonas* | 0.373 |  |  |
| *Libanicoccus* | 0.372 |  |  |
| *Oscillibacter* | 0.366 |  |  |
| *Candidatus.Symbiothrix* | 0.357 |  |  |
| *Enterococcus* | 0.344 |  |  |
| *Lawsonibacter* | 0.344 |  |  |
| *Sulfobacillus* | 0.342 |  |  |
| *Galliscardovia* | 0.341 |  |  |
| *Arabia* | 0.331 |  |  |
| *Eubacterium* | 0.329 |  |  |
| *Schleiferia* | 0.329 |  |  |
| *Desulfofundulus* | 0.326 |  |  |
| *Chlamydia* | 0.323 |  |  |
| *Petrimonas* | 0.323 |  |  |
| *Ralstonia* | 0.315 |  |  |
| *Burkholderia* | 0.315 |  |  |
| *Streptomyces* | 0.310 |  |  |
| *Mogibacterium* | 0.306 |  |  |
| *Megasphaera* | 0.297 |  |  |
| *Arthrobacter* | 0.291 |  |  |
| *Leptolyngbya* | 0.285 |  |  |
| *Butyricimonas* | 0.284 |  |  |
| *Anaerotruncus* | 0.262 |  |  |

**Table S11:** Differential abundant bacterial genera (with FDR correction < 0.05) driving taxa differences between pigs from Ghana and Europe.

| **Driving Ghanaian pigs** | **Effect** | **Driving European pigs** | **Effect** |
| --- | --- | --- | --- |
| *Subdoligranulum* | 7.246 | *Prevotella* | -1.741 |
| *Bifidobacterium* | 2.146 | *Phascolarctobacterium* | -1.606 |
| *Clostridium* | 2.130 | *Sinorhizobium* | -1.193 |
| *Streptococcus* | 2.036 | *Mailhella* | -1.086 |
| *Paenibacillus* | 1.942 | *Campylobacter* | -1.031 |
| *Olsenella* | 1.872 | *Bacteroides* | -0.936 |
| *Clostridioides* | 1.855 | *Brachyspira* | -0.905 |
| *Lactobacillus* | 1.799 | *Faecalibacterium* | -0.879 |
| *Terrisporobacter* | 1.767 | *Anaerovibrio* | -0.828 |
| *Corynebacterium* | 1.741 | *Dyadobacter* | -0.812 |
| *Weissella* | 1.717 | *Paraprevotella* | -0.778 |
| *Staphylococcus* | 1.716 | *Paludibacter* | -0.674 |
| *Bacillus* | 1.704 | *Fibrobacter* | -0.633 |
| *Nocardia* | 1.675 | *Pontibacter* | -0.600 |
| *Romboutsia* | 1.674 | *Acetonema* | -0.596 |
| *Pediococcus* | 1.492 | *Polaribacter* | -0.594 |
| *Nodosilinea* | 1.472 | *Alloprevotella* | -0.575 |
| *Rummeliibacillus* | 1.468 | *Megasphaera* | -0.571 |
| *Halomonas* | 1.432 | *Oxalobacter* | -0.556 |
| *Anoxybacillus* | 1.401 | *Ureaplasma* | -0.551 |
| *Enterobacter* | 1.383 | *Sutterella* | -0.547 |
| *Microcoleus* | 1.380 | *Selenomonas* | -0.544 |
| *Cyanobium* | 1.301 | *Mycoplasma* | -0.512 |
| *Paeniclostridium* | 1.259 | *Spirosoma* | -0.508 |
| *Exiguobacterium* | 1.255 | *Porphyromonas* | -0.504 |
| *Candidatus.Phytoplasma* | 1.242 | *Desulfovibrio* | -0.498 |
| *Escherichia* | 1.208 | *Ruminococcus* | -0.484 |
| *Aerococcus* | 1.193 | *Asteroleplasma* | -0.474 |
| *Lactimicrobium* | 1.187 | *Acidaminococcus* | -0.472 |
| *Collinsella* | 1.109 | *Tannerella* | -0.461 |
| *Shigella* | 1.035 | *Alistipes* | -0.458 |
| *Eggerthella* | 1.027 | *Pseudonocardia* | -0.447 |
| *Enterococcus* | 1.023 | *Mucilaginibacter* | -0.445 |
| *Blautia* | 0.993 | *Dialister* | -0.439 |
| *Akkermansia* | 0.949 | *Spirochaeta* | -0.427 |
| *Acidipropionibacterium* | 0.943 | *Proteus* | -0.420 |
| *Dubosiella* | 0.919 | *Prevotellamassilia* | -0.399 |
| *Listeria* | 0.907 |  |  |
| *Erysipelatoclostridium* | 0.902 |  |  |
| *Mycobacterium* | 0.888 |  |  |
| *Kurthia* | 0.881 |  |  |
| *Adlercreutzia* | 0.863 |  |  |
| *Salmonella* | 0.855 |  |  |
| *Parascardovia* | 0.849 |  |  |
| *Erwinia* | 0.820 |  |  |
| *Lactococcus* | 0.815 |  |  |
| *Galactobacillus* | 0.803 |  |  |
| *Gordonibacter* | 0.790 |  |  |
| *Intestinibacter* | 0.780 |  |  |
| *Klebsiella* | 0.758 |  |  |
| *Lysinibacillus* | 0.744 |  |  |
| *Slackia* | 0.743 |  |  |
| *Actinomyces* | 0.741 |  |  |
| *Turicibacter* | 0.738 |  |  |
| *Erysipelothrix* | 0.732 |  |  |
| *Arthrobacter* | 0.729 |  |  |
| *Anaerolactibacter* | 0.719 |  |  |
| *Viridibacillus* | 0.714 |  |  |
| *Streptomyces* | 0.710 |  |  |
| *Denitrobacterium* | 0.694 |  |  |
| *Micrococcus* | 0.674 |  |  |
| *Dietzia* | 0.673 |  |  |
| *Halobacillus* | 0.671 |  |  |
| *Photobacterium* | 0.661 |  |  |
| *Leifsonia* | 0.650 |  |  |
| *Kandleria* | 0.636 |  |  |
| *Arsenophonus* | 0.635 |  |  |
| *Holdemanella* | 0.632 |  |  |
| *Synechococcus* | 0.627 |  |  |
| *Pseudomonas* | 0.620 |  |  |
| *Serratia* | 0.602 |  |  |
| *Brachybacterium* | 0.593 |  |  |
| *Dickeya* | 0.593 |  |  |
| *Enterorhabdus* | 0.588 |  |  |
| *Paraclostridium* | 0.586 |  |  |
| *Microbacterium* | 0.586 |  |  |
| *Pseudanabaena* | 0.574 |  |  |
| *Acidithiobacillus* | 0.570 |  |  |
| *Pseudoscardovia* | 0.567 |  |  |
| *Oculatella* | 0.555 |  |  |
| *Sharpea* | 0.552 |  |  |
| *Lachnoclostridium* | 0.536 |  |  |
| *Anaerocolumna* | 0.534 |  |  |
| *Sporosarcina* | 0.534 |  |  |
| *Thermotoga* | 0.520 |  |  |
| *Brevibacterium* | 0.497 |  |  |
| *Ochrobactrum* | 0.493 |  |  |
| *Rhodococcus* | 0.469 |  |  |
| *Rathayibacter* | 0.458 |  |  |
| *Libanicoccus* | 0.457 |  |  |
| *Holdemania* | 0.454 |  |  |
| *Kocuria* | 0.453 |  |  |
| *Peptoniphilus* | 0.453 |  |  |
| *Chryseomicrobium* | 0.438 |  |  |
| *Brevibacillus* | 0.437 |  |  |
| *Intestinimonas* | 0.410 |  |  |
| *Acinetobacter* | 0.385 |  |  |
| *Epulopiscium* | 0.375 |  |  |
| *Spiroplasma* | 0.313 |  |  |
| *Acholeplasma* | 0.289 |  |  |

**Figure S1:** Location of the collected pig faecal samples

**
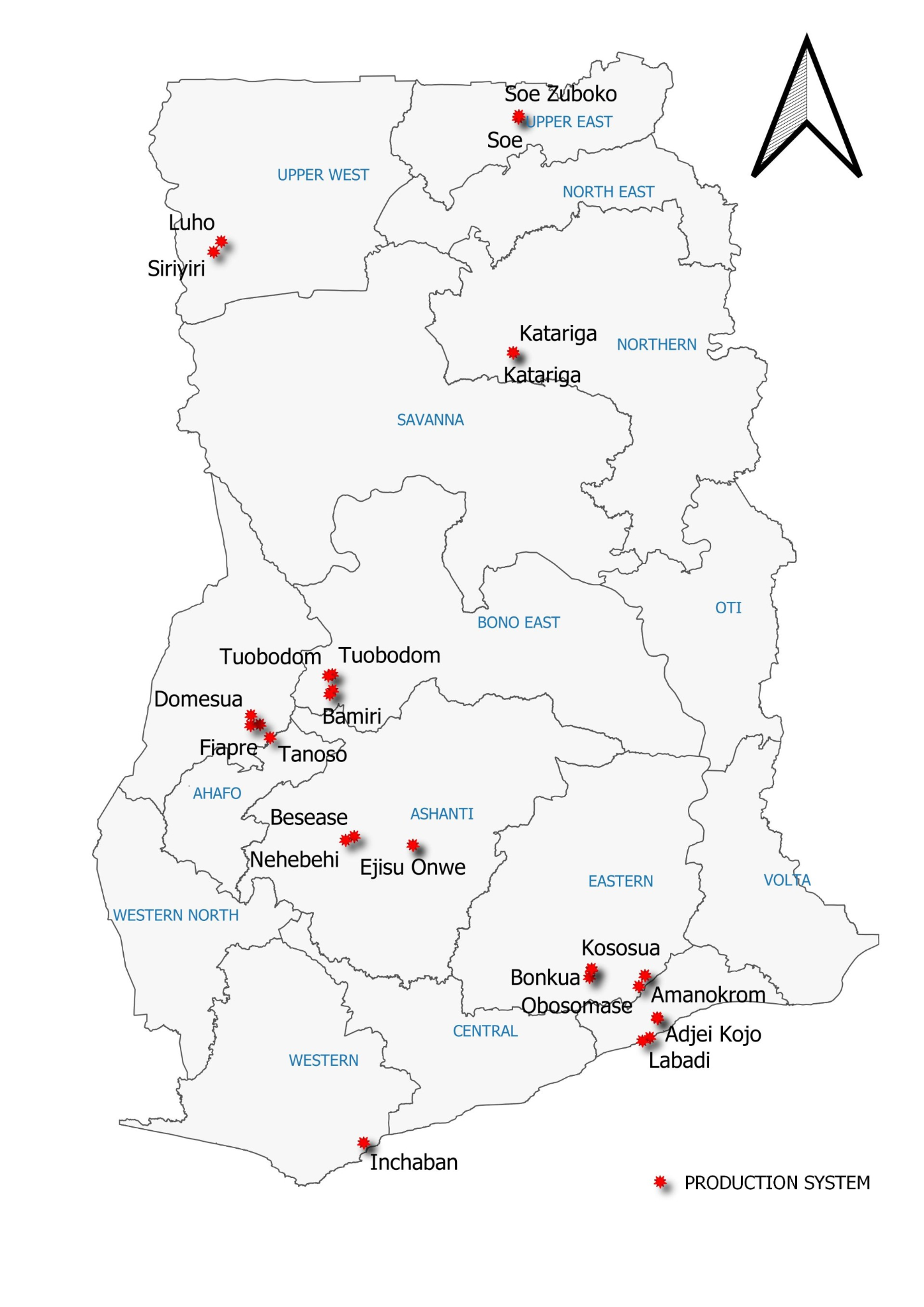
**

**Figure S2:** Size distribution of the fragment counts per sample.


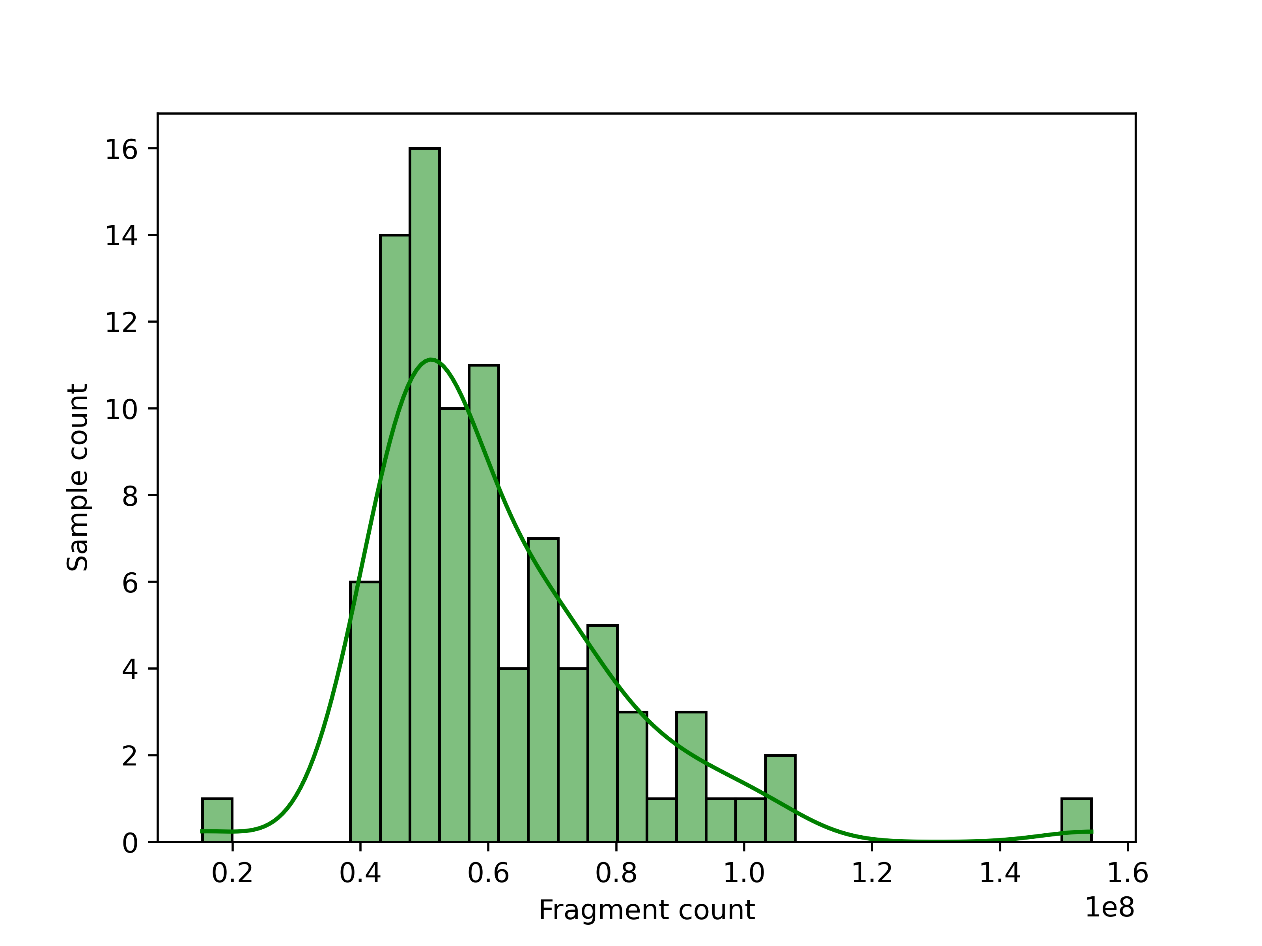


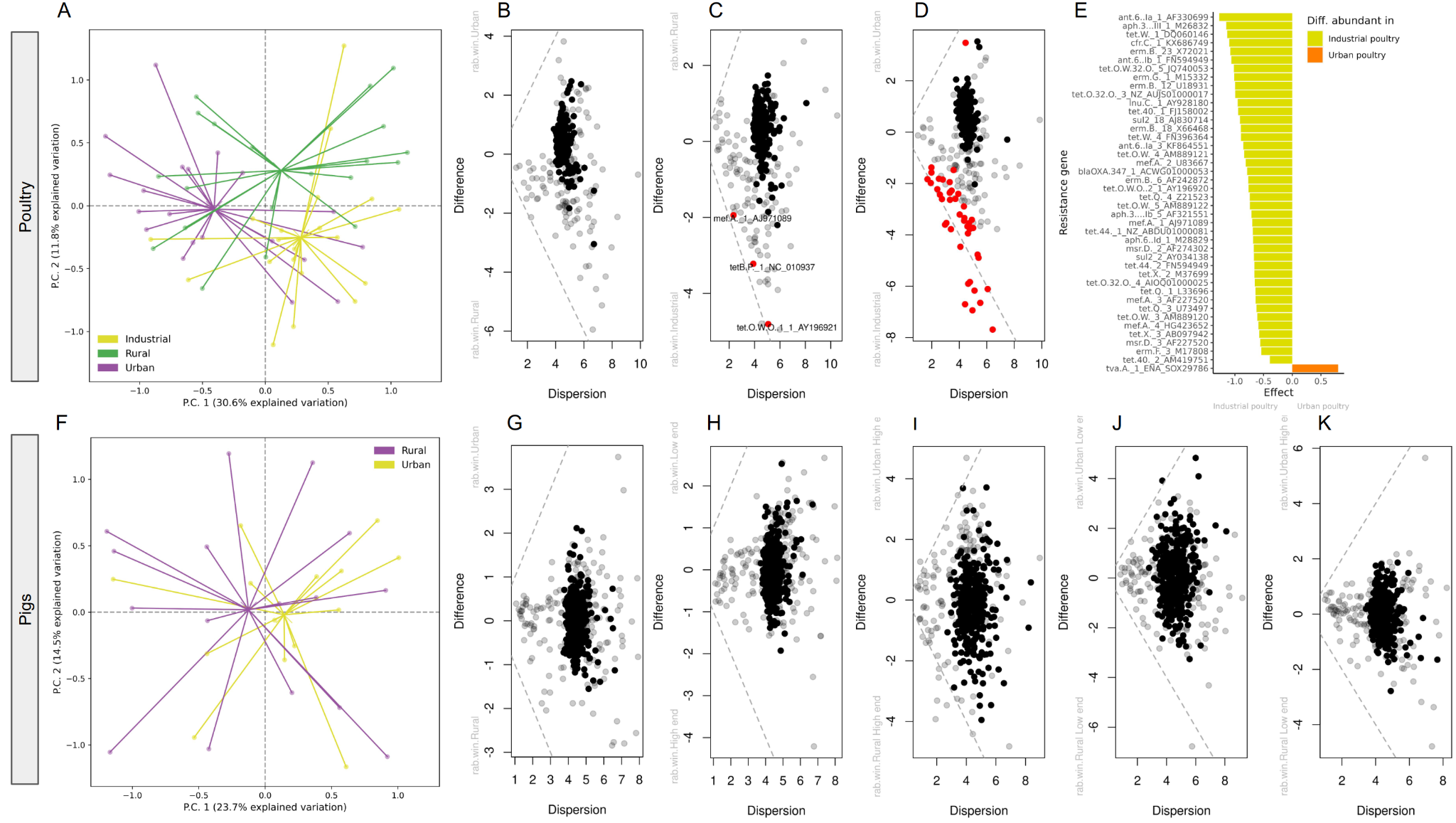


**Figure S3:** Difference in resistance genes within pig populations and poultry populations in Ghana. The ordination analyses (A+F) were performed on the most abundant, most variant centered log-ratio (CLR) transformed size-adjusted counts. For each group, a line connects each sample point to the centroid. **A)** Resistance genes clustering of industrial, urban and rural poultry samples from Ghana. (CLR variance > 2, CLR median > 0.8). **B)** Rural vs urban poultry effect plot from the differential abundance analysis. **C)** Industrial vs rural poultry effect plot. **D)** Industrial vs urban poultry effect plot further investigated in **E)** Statistical significant resistance genes with FDR correction < 0.05 between industrial and urban poultry. **F)** Resistance genes clustering of urban and rural pig samples from Ghana (CLR variance > 3.2, CLR median > 0.5). **G)** Rural vs urban pig effect plot. **H)** High end vs low end biosecurity level pig effect plot. **I)** Rural high end vs urban high end biosecurity level pig effect plot. **J)** Rural low end vs urban low end biosecurity level pig effect plot. **K)** Rural low end vs urban high end biosecurity level effect plot.


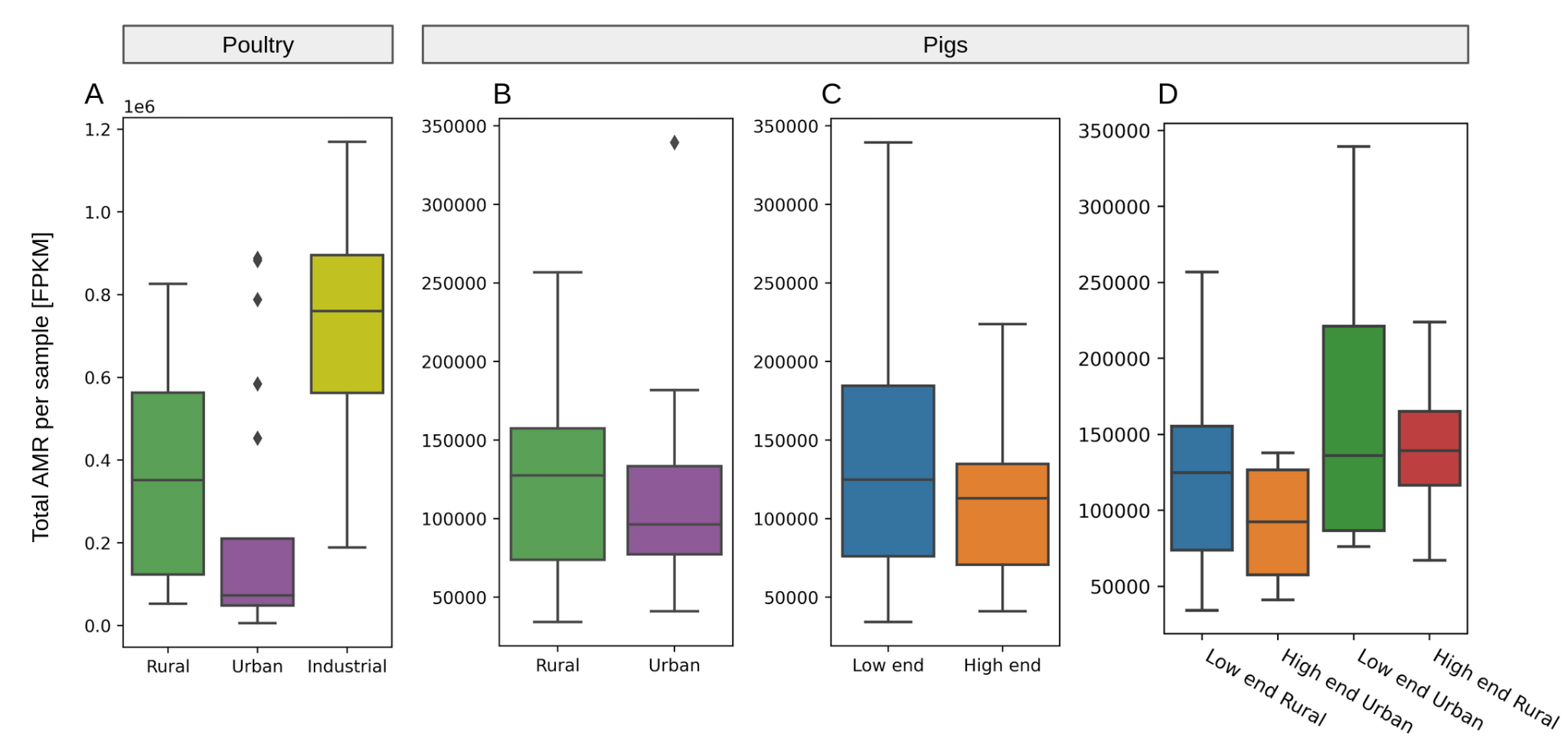


**Figure S4:** Total AMR per sample calculated as the total AMR fragments per kilobase per million fragments per sample (FPKM), stratified by host. **A)** Rural (n=20), urban (n=20) and industrial poultry (n=20) from Ghana. **B)** Urban (n=14) and rural (n=16) pigs from Ghana. **C)** Low-end (n=16) and high-end (n=10) biosecurity pigs from Ghana. **D)** Biosecurity combined with location pigs from Ghana: Rural low-end biosecurity pigs (n=12), urban high-end biosecurity pigs (n=10), urban low-end biosecurity pigs (n=4) and rural high-end biosecurity pigs (n=4).


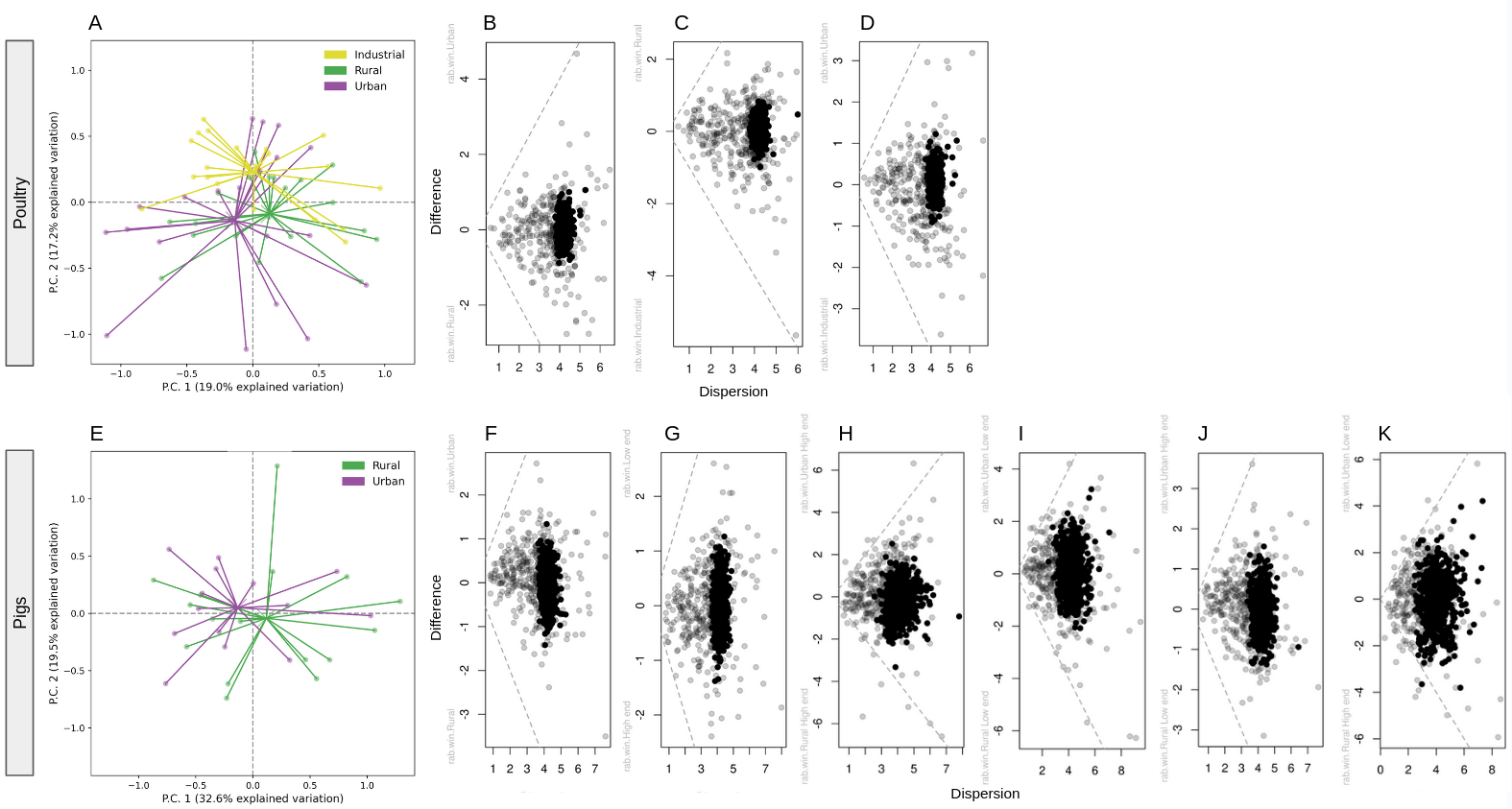


**Figure S5:** Difference in bacterial genera within pig populations and poultry populations in Ghana. The ordination analyses (A+E) were performed on the most abundant, most variant centered log-ratio (CLR) transformed size-adjusted counts. For each group, a line connects each sample point to the centroid. The effect plots (B-D+F-K) from the differential abundance analysis shows the within-group dispersion of CLR values of each bacteria genus compared to the between-group differences. Statistical significant resistance genes with a Benjamini-Hochberg false-discovery rate (FDR) correction < 0.05 are colored red. The gray dotted line indicates an effect size of 1. **A)** Bacterial genera PCA clustering of poultry samples (CLR variance 1.5, CLR median > 0). **B)** Effect plot comparing rural vs urban poultry. **C)** Effect plot comparing industrial vs rural poultry. **D)** Effect plot comparing industrial vs urban poultry. **E)** Bacterial genera PCA clustering of pig samples (CLR variance > 2.5, CLR median > 0). **F)** Effect plot comparing rural vs urban pigs. **G)** Effect plot comparing high end biosecurity level pigs vs low end biosecurity level pigs. **H)** Effect plot comparing rural high end biosecurity level pigs vs urban high end biosecurity level pigs. **I)** Effect plot comparing rural low end biosecurity level pigs vs urban low end biosecurity level pigs. **J)** Effect plot comparing rural low end biosecurity level pigs vs urban high end biosecurity level pigs. **K)** Effect plot comparing rural high end biosecurity level pigs vs urban low end biosecurity level pigs.


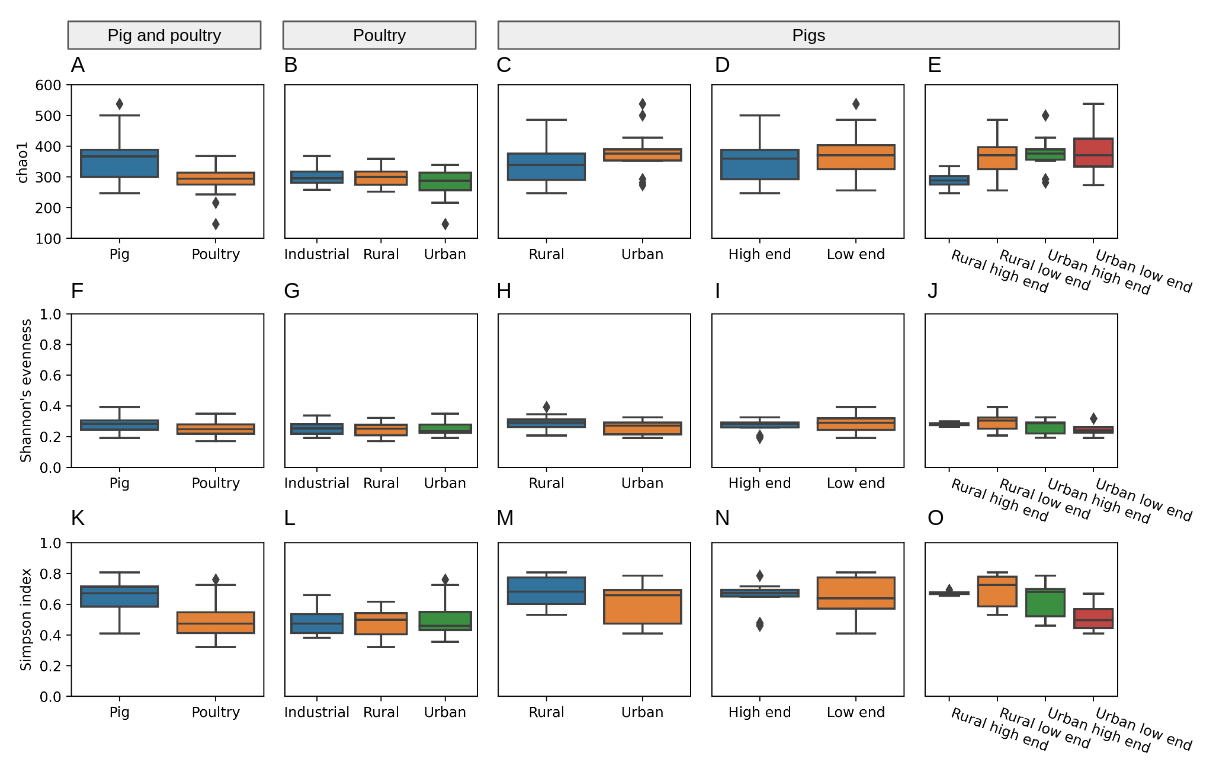


**Figure S6:** Alpha-diversity of bacterial genera in pigs (n=30), poultry (n=60), industrial poultry (n=20), urban poultry (n=20), rural poultry (n=20), urban pigs (n=14), rural pigs (n=16), high-end biosecurity pigs (n=14), low-end biosecurity pigs (n=16), urban high-end biosecurity pigs (n=10), rural low-end biosecurity pigs (n=12), rural high-end biosecurity pigs (n=4) and urban low-end biosecurity pigs (n=4) from Ghana. **A-E)** chao1 richness. **F-J)** Shannon’s evenness. **K-O)** Simpson index.


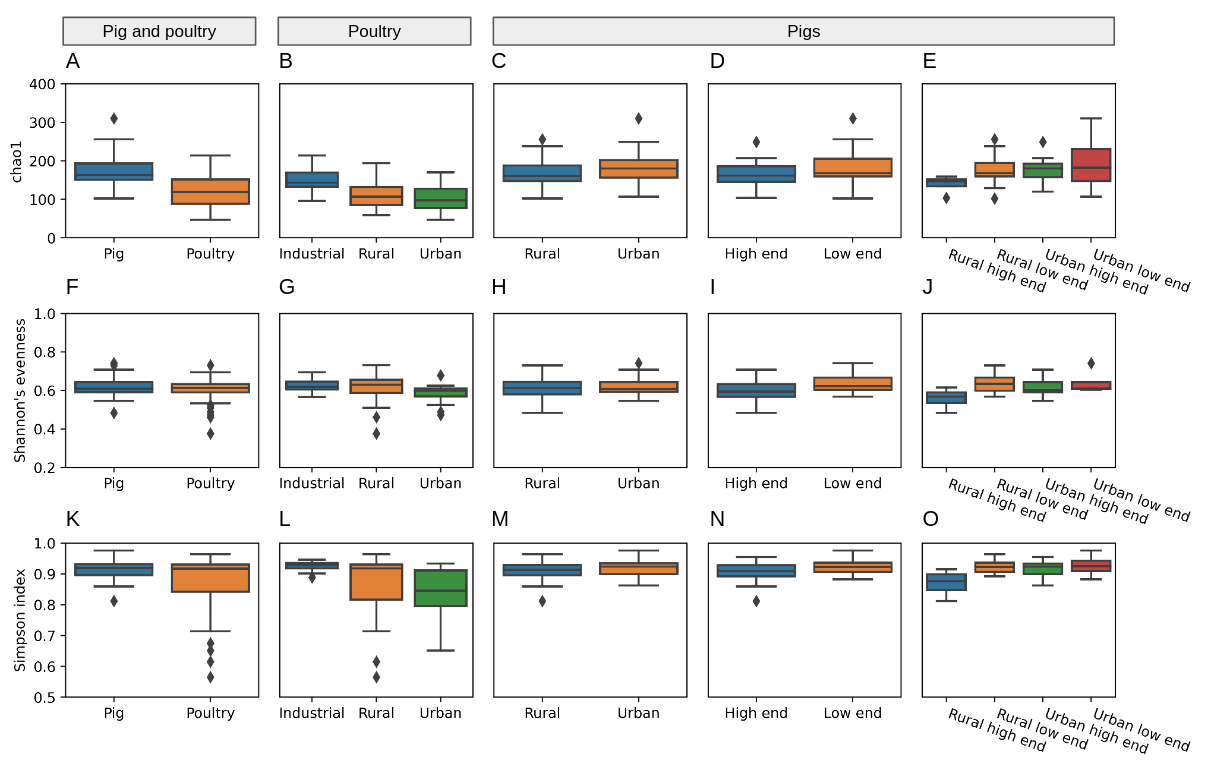


**Figure S7:** Alpha-diversity of resistance genes in pigs (n=30), poultry (n=60), industrial poultry (n=20), urban poultry (n=20), rural poultry (n=20), urban pigs (n=14), rural pigs (n=16), high end biosecurity pigs (n=14), low end biosecurity pigs (n=16), urban high end biosecurity pigs (n=10), rural low end biosecurity pigs (n=12), rural high end biosecurity pigs (n=4) and urban low end biosecurity pigs (n=4) from Ghana. **A-E)** chao1 richness. **F-J)** Shannon’s evenness. **K-O)** Simpson index.
